# Oncolytic Viruses—Natural and Genetically Engineered Cancer Immunotherapies

**DOI:** 10.3389/fonc.2017.00202

**Published:** 2017-09-11

**Authors:** Sachin R. Jhawar, Aditya Thandoni, Praveen K. Bommareddy, Suemair Hassan, Frederick J. Kohlhapp, Sharad Goyal, Jason M. Schenkel, Ann W. Silk, Andrew Zloza

**Affiliations:** ^1^Rutgers Cancer Institute of New Jersey, New Brunswick, NJ, United States; ^2^Department of Radiation Oncology, Rutgers Robert Wood Johnson Medical School, Robert Wood Johnson University Hospital, New Brunswick, NJ, United States; ^3^Department of Pathology, Brigham and Women’s Hospital, Boston, MA, United States; ^4^Department of Medicine, Rutgers Robert Wood Johnson Medical School, New Brunswick, NJ, United States; ^5^Department of Surgery, Rutgers Robert Wood Johnson Medical School, New Brunswick, NJ, United States

**Keywords:** oncolytic viruses, cancer immunotherapy, oncoimmunology, pathogens, viruses

## Abstract

There has long been interest in innovating an approach by which tumor cells can be selectively and specifically targeted and destroyed. The discovery of viruses that lyse tumor cells, termed oncolytic viruses (OVs), has led to a revolution in the treatment of cancer. The potential of OVs to improve the therapeutic ratio is derived from their ability to preferentially infect and replicate in cancer cells while avoiding destruction of normal cells surrounding the tumor. Two main mechanisms exist through which these viruses are reported to improve outcomes: direct lysis of tumor cells and indirect augmentation of host anti-tumor immunity. With these factors in mind, viruses are chosen or modified to selectively target tumor cells, decrease pathogenicity to normal cells, decrease the antiviral immune response (to prevent viral clearance), and increase the antitumor immune response. While only one OV has been approved for the treatment of cancer in the United States, and only two other OVs have been approved worldwide, a wide spectrum of OVs are in various stages of preclinical development and in clinical trials. These viruses are being studied as alternatives and adjuncts to more traditional cancer therapies including surgical resection, chemotherapy, radiation, hormonal therapies, targeted therapies, and other immunotherapies. Here, we review the natural characteristics and genetically engineered modifications that enhance the effectiveness of OVs for the treatment of cancer.

## Introduction

The implementation of immunotherapeutic strategies for the treatment of cancer has gained prominence over the past decade in preclinical development and clinical practice. Traditional oncological approaches, including surgery, radiation, and chemotherapy, aim to directly remove or kill cancer cells. In contrast, immunotherapy seeks to enhance the host immune system’s ability to eliminate cancer cells resulting in tumor regression, antitumor immune memory formation, and ultimately in durable responses (1, 2). Induction of the host immune system *via* increases in innate and adaptive immune surveillance of and response against the tumor can provide lasting positive outcomes in cancer patients (3). Initiating the human body’s ability to recognize and destroy malignancies is often better tolerated by patients long term in comparison to traditional therapies. However, the use of immunotherapies in conjunction with traditional therapies may further increase treatment efficacy and lead to prolonged survival (2, 4–6). Immunotherapies have also been shown to be effective against recalcitrant disease and are, therefore, being tested and utilized frequently in difficult clinical situations for patients with advanced-stage disease. Delivering immunotherapy to patients with earlier stage cancers may lead to an increase in the proportion of patients who exhibit clinical benefit.

A novel addition to the anticancer treatment armamentarium is use of oncolytic viruses (OVs). Observations of spontaneous tumor regression after naturally occurring viral infections gave rise to the notion that OVs can be incorporated as treatments (7). Although numerous naturally occurring OVs exist, recently immense interest has revolved around genetically modifying viruses to create new cancer therapeutics (8, 9). OVs function by preferentially targeting and killing tumor cells while simultaneously stimulating the immune system and creating antitumor immunity (4–6). This dual mechanism of action allows for direct local antitumor response (leading to tumor regression) as well as the induction of the innate and adaptive components of the immune system (leading to the recognition and removal of systemic disease and prevention of recurrence) (7). The predilection of these viruses to preferentially infect tumor cells while sparing normal surrounding cells allows for an excellent therapeutic ratio. The ability of OVs to cause immune infiltration into tumors bridges the gap to immunotherapy (1, 4–6), and immunologic outcomes are being reported from ongoing and completed clinical trials (Table 1).

**Table 1:** Select clinical trials of oncolytic viruses with clinical and immune outcomes data (9–17).

Virus strain	Study	Trial design	Number of patients	Clinical outcomes	Immunological outcomes
Herpes simplex virus (HSV) type 1 (HSV-1)	Talimogene Laherparepvec Improves Durable Response Rate in Patients with Advanced Melanoma (9)	Phase III	436	Improved durable response rate (16.3 vs. 2.1%), overall response rate (26.4 vs. 5.7%), and longer median survival (23.3 vs. 18.9 months) in patients with non-surgically resectable melanoma receiving T-VEC vs. GM-CSF (9)	Regression of 34% of uninjected non-visceral and 15% of visceral tumors (11). Earlier Phase II study reported increased MART-1 specific T cells in regressing tumors and decreases in intratumoral regulatory T cells, suppressor T cells, and myeloid-derived suppressor cells in responding patients (12)
HSV-1	Talimogene Laherparepvec in Combination with Ipilimumab in Previously Untreated, Unresectable Stage IIIB-IV Melanoma (10)	Phase Ib/II	19	50% objective response rate and 44% of patients had a durable response lasting ≥6 months. 18-month progression-free survival was 50%, and overall survival was 67%	Significant increase in total CD8+ T cells and activated CD8+ T cells (CD3+, CD4−, HLA-DR+). Significant upregulation in activation marker ICOS on CD4+ T cells
Reovirus	Randomized Phase II Trial of Oncolytic Virus Pelareorep (Reolysin) in Upfront Treatment of Metastatic Pancreatic Adenocarcinoma (13)	Phase II	73	Addition of pelareorep to carboplatin and paclitaxel did not improve progression-free survival compared to carboplatin and paclitaxel alone	Increased natural killer cells or B cells in patients with improved disease control rate
Reovirus	Phase II trial Intravenous Administration of Reolysin (Reovirus Serotype-3-Dearing Strain) in Patients with Metastatic Melanoma (14)	Phase II	21	No objective responses seen and 6 patients with stable disease for >8 weeks	Extensive necrosis in metastases of one patient and demonstrated viral replication in melanoma metastases in 2 of 13 tumors. Significant increase in neutralizing anti-reovirus titers in 13 patients
Vaccinia virus	Use of a Targeted Oncolytic Poxvirus, JX-594, in Patients with Refractory Primary or Metastatic Liver Cancer: A Phase I Trial (15)	Phase I	14	JX-594 injection was generally well tolerated. Neutralizing antibodies do not prevent efficacy	Interleukin 6, Interleukin 10, and TNF-α peaks at 3 h and at later time points (days 3–22)
Coxsackievirus	Phase II Calm Extension Study: A Study of Intratumoral CAVATAK™ in Patients with Stage IIIc and Stage IV Malignant Melanoma (16)	Phase II	57	38.6% of evaluable patients demonstrated durable responses in both injected and uninjected melanoma metastases	Increased immune-cell infiltration (in particular CD8+ cells) and increased PD-L1 expression on immune cells. Gene expression analysis 4 days pre and post biopsy samples indicated Th-1 gene shift
Coxsackievirus	Phase Ib Study of Intratumoral Oncolytic Coxsackievirus A21 (CVA21) and Systemic Pembrolizumab in Subjects with Advanced Melanoma: Interim results of the CAPRA clinical trial (17)	Phase Ib	22	Best overall response rate of 60% and stable disease in 26.7% of patients	Increase in number of PD-L1-expressing immune cells and increase in CD8+ and CD4+ T cells observed 8 days post treatment

As our understanding of cancer biology and virus–host cell interactions improves in concert with genetic engineering, the ability to manipulate the viral genome gains importance. For example, talimogene laherparepvec (T-VEC) is a herpes simplex virus (HSV) type 1 (HSV-1) that is the first OV to be approved by the FDA for the treatment of advanced melanoma (9, 18). Although derived from a naturally occurring HSV strain, it has been genetically engineered to preferentially target cancer cells and to result in the production of an immune factor, granulocyte macrophage colony-stimulating factor (GM-CSF), to improve the antitumor immune response. T-VEC is now being tested in other cancers and in rational combinations with standard and immune-targeted therapies (9, 18).

**Figure 1:**
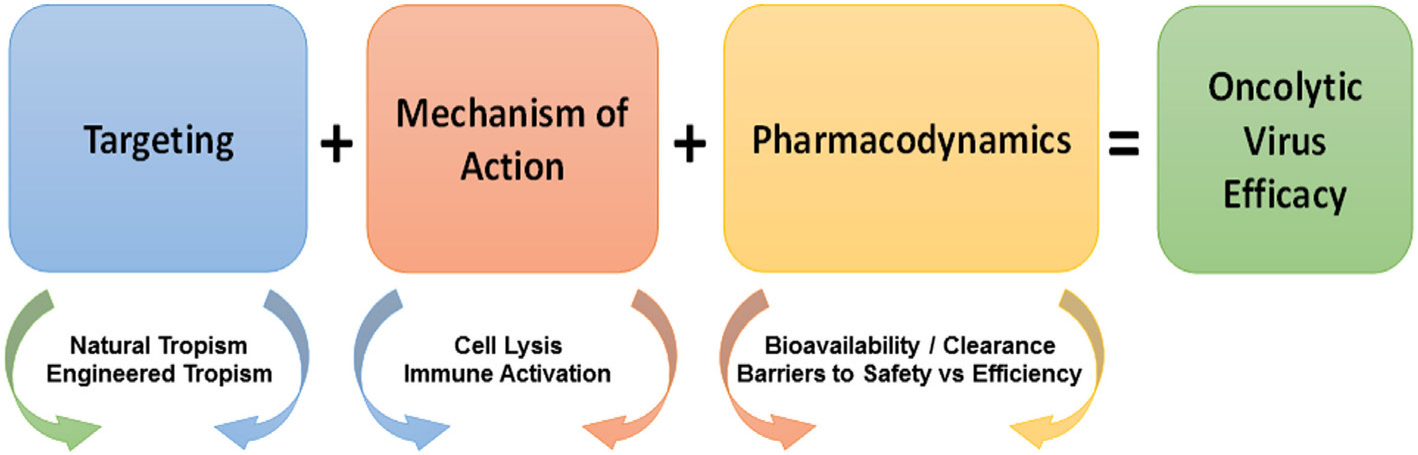
Considerations in the development of oncolytic viruses (OVs). Considerations in the development of efficacious OV immunotherapy include targeting, mechanism of action, and pharmacodynamics. Targeting (blue box) is dependent on the natural and engineered tropism of viruses for tumor vs. normal cells. The mechanism of action (red box) of OVs is dependent on the immune mechanisms and the non-immune mechanisms of OVs, which are further enhanced by the combination of OVs with traditional and emerging antitumor therapeutics. OVs share pharmacodynamic considerations (orange box) with other small molecule drugs as well as raise new fundamental issues in terms of bioavailability vs. clearance and barriers to safety vs. efficiency. Overlapping arrow colors signify the existent overlap between the listed considerations.

This review will describe a series of OVs used in clinical trials and care settings, as well as, in testing and development. It will especially focus on mechanisms underlying the oncolysis of naturally occurring OVs and the genetic modifications that have been made to improve the therapeutic ratio provided by OVs. A number of important considerations exist in terms of choosing a virus for potential use as a therapeutic oncolytic agent. These include targeting the OV to the appropriate tissue or cell type and mechanisms of action (including specific lysis of tumor cells and the activation of an effective immune response) (Figure 1). These are described in this review, while other considerations, including bioavailability and safety have been described in detail in our earlier review (19). Ultimately, this review will address the quickly progressing field of OVs and their role in the fields of oncology and immuno-oncology.

## Targeting

### Targeting: Natural

**Figure 2:**
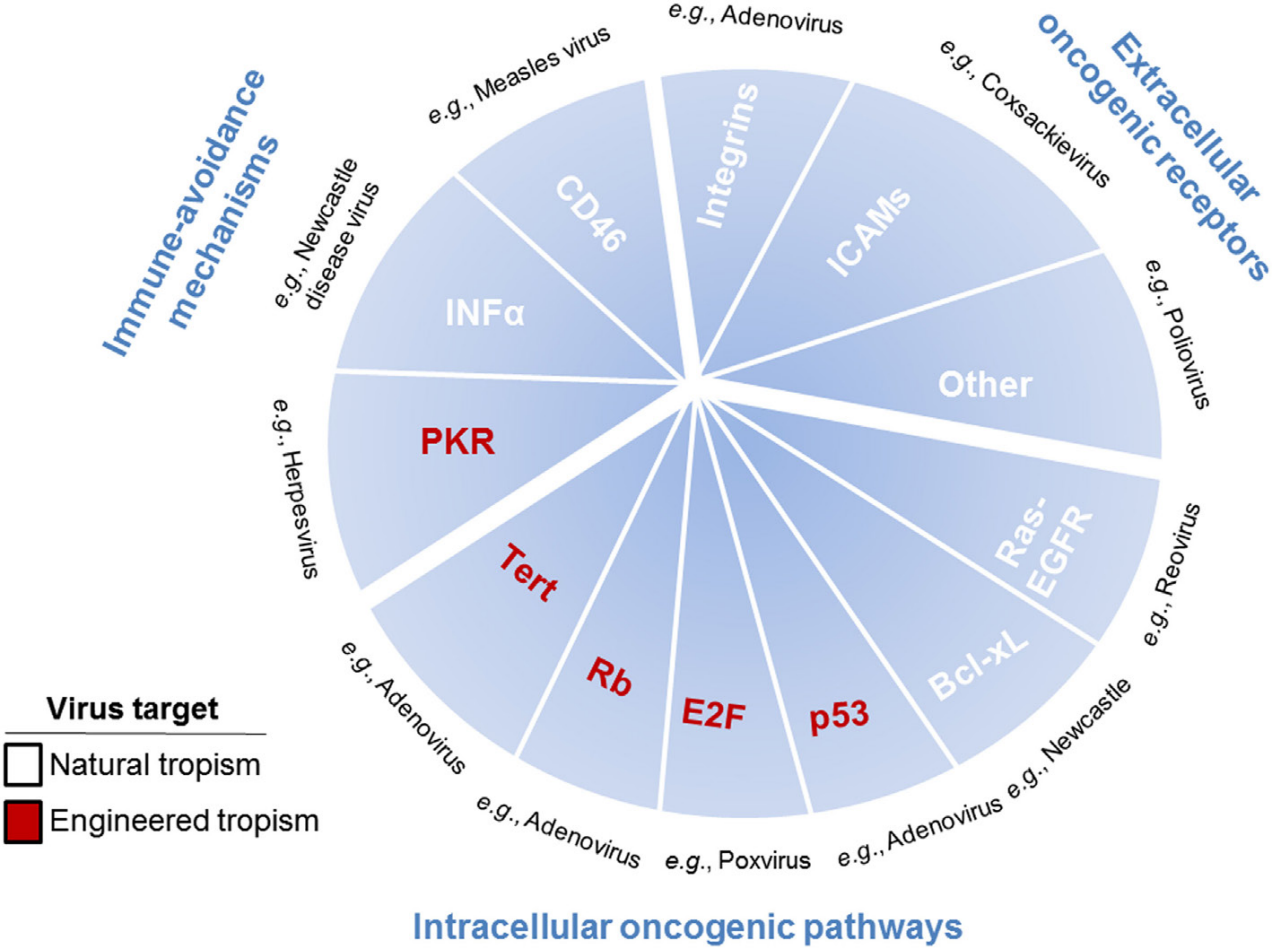
Pathways, receptors, and mechanisms used by oncolytic viruses (OVs) to target cancer cells. OVs target cancer cells through pathways, receptors, and mechanisms used to promote tumor growth, including immune-avoidance mechanisms, extracellular oncogenic receptors, and intracellular oncogenic pathways. Immune-avoidance, extracellular receptor, and intracellular pathway targets that are overexpressed or repressed to inherently allow tumors to avoid immune responses can simultaneously can be used for targeting OVs to cancer cells. Extracellular receptors include surface molecules [such as integrins, ICAMs, and others (CD155, laminin receptors, etc.)] inherently expressed by some tumor cells, which are utilized by viruses for specific targeting to cancer cells rather than normal cells. Intracellular pathways are utilized in tumor cells to promote proliferation and survival, which are required for viral propagation, thus enhancing cancer cell susceptibility to oncolytic viral infection. Single examples of viruses utilizing each of the described targets are listed in black text. Mechanisms, pathways, and receptors that enhance cancer cell targeting as part of the natural tropisms of OVs are listed in white text and as part of the engineered tropisms of OVs are listed in red text.

In the development of cancer therapeutics, targeting may be the most difficult obstacle to overcome. Targeting can be achieved by choosing viruses with natural tropisms to specific tissues or cell types or by engineering these tropisms (Figure 2). While research studies tend to highlight differences between tumor cells and normal cells, in reality their similarities are much more numerous. This presents the problem of specific drug targeting that allows for destruction of the tumor without effecting normal (i.e., non-cancerous) tissue. OVs present two distinct, although not mutually exclusive, advantages by which they can specifically target cells. The first is the basic tenet of viral infection, that viruses naturally exploit permissive cells for infection through expression of the necessary surface receptors that allow viral entry and through the modulation of host defense pathways that allow viruses to avoid detection. The second is the permissiveness of viruses to accept modifications engineered to increase their specificity against cancer cells, while at the same time being limited in their effect on normal cells.

Cell permissivity for viral infection begins with the ability of the virus to identify and enter its cellular target. This is mediated primarily through the expression of cell surface receptors. Several OVs in use have been chosen based on their inherent ability to use cell surface molecules for entry that are abnormally upregulated in cancer cells. An example of this is the use of HVEM, nectin-1, and nectin-2 by T-VEC (20). This augmented expression has been noted in multiple tumor types and renders these cancer cells more susceptible to herpesvirus infection than normal cells. Another example of cancer cell surface molecule-specific tropism includes CD46. This molecule is aberrantly expressed by tumor cells to subvert the complement pathway and is frequently overexpressed by malignant cells to avoid recognition and elimination. This can be taken advantage of by use of the measles virus (Edmonston strain) which utilizes CD46 for cell entry, making tumor cells overexpressing this receptor susceptible to oncolysis (21). Similarly, overexpression of intracellular adhesion molecule 1 (ICAM-1) and decay accelerating factor in malignancies such as breast cancer, multiple myeloma, and melanoma can be taken advantage of by coxsackievirus, including coxsackievirus A21 (CAVATAK) (22–24). A series of other OVs are being developed for clinical use based on their natural tropism for cancer cells. For example, poliovirus has a natural tropism for the cell surface marker CD155. This receptor is frequently upregulated by cancers because it affords protection from innate immunity for the pathogen by downregulating antitumor natural killer (NK) cell responses (25). Echovirus, another enterovirus, has tropism for ovarian cancer because of higher expression of integrin α2β1 (26). Besides making use of cell surface receptors designed to subvert the immune system, some OVs use receptors utilized by tumor cells to enhance metastasis. Cancer cells may upregulate the laminin receptor to allow for invasion and increased motility. The Sindbis virus targets this receptor, allowing for specific targeting of cells with increased metastatic potential (27).

Cancer cells use more than aberrant cell surface receptor expression to fuel carcinogenesis. Specifically, they manipulate the transcriptional and signaling networks of cells to increase survival, proliferation, immune evasion, and metastasis. Viruses use many of these same pathways to propagate and infect cells. These parallels lead to cancer cells being more permissible to viral infections based on the overlaps between carcinogenesis and viral infection. An example is cancer’s resistance to cell death, often achieved by increased expression of antiapoptotic molecules such as those of the Bcl family. This increase in antiapoptotic molecules increases the targeting of cancer cells by certain OVs. Newcastle disease virus, for example, specifically targets cancer cells overexpressing Bcl-xL, a protein that inhibits apoptosis. This gives the virus time to incubate, multiply, and form syncytia, which is imperative to the survival and spread of Newcastle disease virus (28). The Ras signaling pathway represents another pathway altered in cancer cells. Ras is involved in the regulation of cell death and proliferation. Reovirus and vaccinia have been identified as OVs due to their ability to specifically target cancer cells driven by the Ras pathway. Reovirus preferentially proliferates in Ras-transformed cells (29). Reovirus infects healthy cells and begins viral RNA production. This viral RNA causes activation of natural cellular defenses including the PKR pathway, which leads to translation inhibition, thus stopping viral production and spread. In cancer cells in which this pathway has been manipulated; however, the PKR pathway is not activated, which allows increased viral production and lysis (29). Vaccinia virus, a pox family virus, targets malignancies that overexpress EGFR. It requires EGFR-RAS signaling to replicate. These viruses encode a ligand that can bind to the EGFR receptor, resulting in RAS activation. This leads to the increased production of virus and propagation of the infection (30).

Further, a number of viruses have been identified and taken to clinical trial that are oncolytic through preferentially targeting transformed cells by mechanisms that have not yet been fully elucidated. For example, Seneca valley virus is being clinically evaluated for its therapeutic efficacy against neuroendocrine tumors based on its natural tropism for cancer cells with neuroendocrine features (31, 32). One possibility is that it utilizes targets (including CD56, chromogranin A, and potentially synaptophysin) that are associated with neuroendocrine tumors. Another example is parvovirus H-1PV, which preferentially targets glioblastoma, pancreatic carcinoma, and other tumors through complex mechanisms that are not yet fully understood. One possibility is that parvoviruses may target transformed cells through increased cellular proliferation allowing for production of viral DNA and proteins, including those viral components that are needed for lysis of the cell (33). For these viruses and others, it may also be that they require cellular proliferation and that cancer, a disease of abnormally elevated proliferation, creates a natural tropism for OVs, thus providing a means for therapeutic targeting of cancer.

### Targeting: Engineered

Advances in molecular biology have afforded the OV field an opportunity to alter the DNA sequences of viruses and thus engineer viruses that are more specific for cancer cells than their normal counterparts. Currently, in clinical trials and in preclinical development, such viruses employ multiple mechanisms, including expression of modified receptors for cellular entry, restriction of critical viral protein expression *via* cancer-specific promoters, and deletion of viral proteins that prevent apoptosis in healthy cells.

To target ovarian cancer, an oncolytic adenovirus was engineered so that its capsid incorporated a specific arginine–glycine–aspartic acid (RGD) protein motif so that it could bind to α_v_β_3_ and α_v_β_5_ (34) since these cell surface receptors are overexpressed in ovarian cancer (35). Similarly, to allow for targeting of melanoma by a lentivirus, the E2 glycoprotein of the Sindbis virus, which has a natural affinity to these cells, has been expressed (36). This establishes the concept that OVs may be able to be engineered to directly target cancer cells based on unique cell surface receptors.

Cancer cells inherently have altered signaling pathways that allow for uncontrolled proliferation. Molecular biology is allowing viruses to be modified and provided tropisms that take advantage of protein regulation and signaling in cancer cells. One technique used is the manipulation of viral genes so they are under the control of modified promoters. Adenoviruses are commonly engineered in this manner based on their large viral genome, which allows for the incorporation of long DNA sequences thus permitting multiple modifications to be made to the native virus. E1A is an adenoviral protein that inhibits retinoblastoma (Rb)-mediated cell cycle arrest, allowing for sustained viral replication. It has been manipulated in various ways to allow for selective viral tropism in tumor cells. In an engineered virus specific to prostate cancer, for example, this gene has been modified to be under a prostate-specific antigen (PSA) promoter. PSA is a protein that is created specifically in normal and malignant prostate cells. This modification leads to E1A expression, and therefore, viral proliferation and oncolysis, that only occurs in prostate cells (37). In other cells, however the adenovirus will not produce E1A and therefore Rb-induced apoptosis will occur normally, thus halting infection. The KH901 virus is also a modified adenovirus that expresses E1A in actively dividing cells. This is achieved by having E1A transcription coupled to human telomerase reverse transcriptase (hTERT) and further being restricted by the presence of E2F-1 (cell cycle regulator) on the hTERT promoter (38). Here, using the human telomerase promoter increases the number of different tumors susceptible to this adenovirus. E1A manipulation has also been used in the development of CG0070. This virus has been modified to selectively replicate in cells that have an Rb deficiency or defect. Normally, Rb binds to E2F, inhibiting this factor’s activity and its ability to conjugate with other molecules (39). In the context of Rb depletion, E2F is free to bind. Therefore, the E1A protein has been placed under the control of the E2F-1 promoter in this virus (40). Again, multiple cancers are susceptible because E2F-1 regulation of E1A expression is restricted to cells with defective Rb, which is a common mutation in cancers. The tumor microenvironment is often hypoxic, protecting the tumor cells from traditional therapies, such as radiation, that require oxygenation to work optimally. However, OVs with A1E manipulation can be engineered to take advantage of such an environment. Specifically, HYPR-Ad is an adenovirus whose E1A expression is transcriptionally regulated by HIF-1α, a protein induced by the hypoxic environment (41).

In addition to restricting viral replication to tumor cells, adenoviruses have been engineered to use targeted delivery of suicide genes with promoters that have increased activity. One example is the placement of the HSV-1 thymidine kinase (TK) suicide gene under the control of an osteocalcin promoter. Osteocalcin is overexpressed in patients with bone metastases. This modification restricts the toxicity of the suicide gene to cells with an active osteocalcin promoter and increases susceptibility of cancer cells with overactive osteocalcin promoters (42). This strategy of targeting cancer cells by using promoters that are tissue-specific or enriched in the tumor limits the effects of the viruses to these areas, which can improve the therapeutic ratio by limiting side effects spatially.

To promote tumorigenesis, cancer cells disrupt natural immune system antiviral defenses and thus do not function like normal cells. This allows for the engineering of viruses to induce proliferation and lysis of cancer cells while inducing apoptosis in normal cells. Adenoviruses, for example, normally act to prevent abortive apoptosis in normal cells through the E1B protein, which binds to and inactivates the p53 protein. OVs ONYX-15 and H101 have been engineered to prevent expression of E1B (43). While these viruses retain the ability to infect normal cells, they will not actively replicate in them because the cells will undergo apoptosis, thus stopping the spread of the virus to other nearby normal cells. Cancer cells, on the other hand, that downregulate or inactivate p53, will remain susceptible to viral replication and lysis, and allow the spread of the virus to other cancer cells.

Other viruses, including oncolytic herpesvirus have been likewise engineered to take advantage of the disruption of standard antiviral responses that are inherent to cancer cells. Cancer cells commonly have overactive Ras-MAPK signaling which blocks phosphorylation of PKR. Such PKR blockade permits cellular proliferation. The HSV JS1 strain (the backbone of T-VEC) has been engineered with deletions of ICP 34.5 and US11. ICP34.5 acts as a neurovirulence factor required for viral replication that works by interacting with PCNA, and the US11 gene product downregulates expression of PKR. These virulence factors are required in normal infection, but since tumor cells undergo aberrant division and frequently downregulate PKR, this attenuated virus is able to preferentially infect cancer cells (44).

## Direct Cell Lysis

### Cell Lysis: Natural

**Figure 3:**
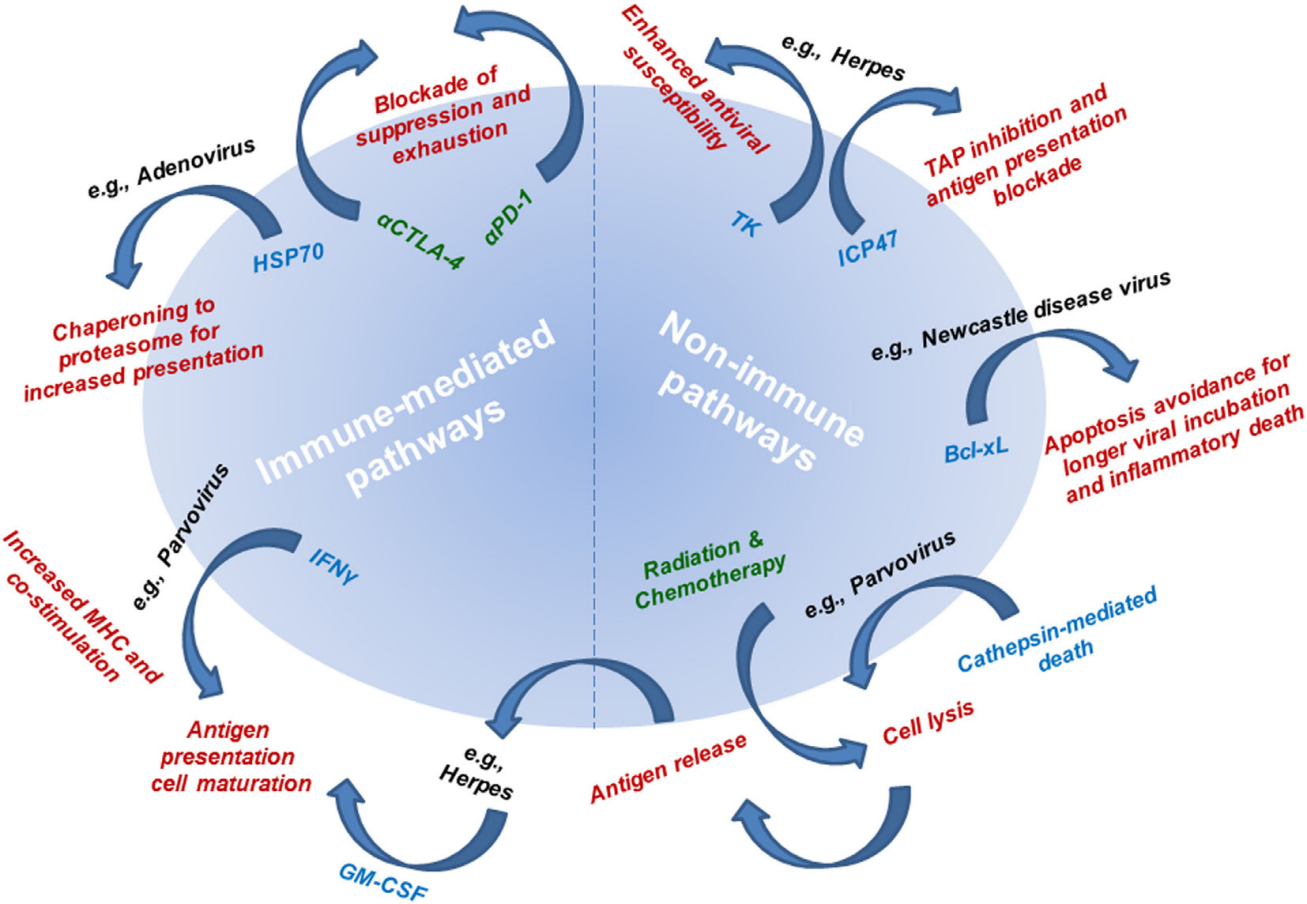
Mechanisms of action underlying the efficacy of oncolytic viruses (OVs). Mechanistic pathways are categorized as immune-mediated and non-immune pathways. Non-immune pathways are utilized by OVs to avoid apoptotic (non-immunogenic) death to allow sufficient viral infectivity and spread, while at the same time resulting in cell lysis. Connecting non-immune and immune pathways is antigen release as a result of viral infection-induced cell lysis. The mechanism underlying immune-mediated pathways includes delivery or utilization of immune mediators that allow for improved tumor antigen presentation and subsequent tumor-targeted immune responses. Radiation therapy and chemotherapy (leading to cell lysis and antigen release) and immunotherapy (leading to blockade of antitumor response suppression and exhaustion) are being combined with OVs to simultaneously target multiple mechanistic pathways for improved antitumor therapeutic responses. Mechanisms of action are listed in red text. Single examples of viruses utilizing each of the described mechanisms are listed in black text next to their respective mechanism. Mediators responsible for each mechanism are listed in blue text next to their respective mechanism. Therapies used in combination regimens with OVs are listed in green text.

The first-line mechanism of action of OVs is their direct lysis of cancer cells (Figure 3). Some OVs take advantage of dysregulate apoptotic pathways in cancer cells to shunt cells toward other forms of death. One example of this is parvovirus H-1PV, which is currently being evaluated in a phase I/IIa clinical trial for metastatic inoperable pancreatic ductal adenocarcinoma (PDAC), while the first parvovirus clinical trial in patients with progressive recurrent glioblastoma has been recently completed. Toward prolonged survival, many tumors, including glioma and PDAC, actively dysregulate apoptotic pathways, which are inhibited even in the context of viral infection. Thus, H-1PV utilizes the cathepsin-mediated pathway to cause tumor cell death (45, 46). This type of non-apoptotic death is immunogenic and through a bystander effect, leads to an increase of interferon (IFN)-γ, inflammatory cytokines, and tumor neoantigen exposure from oncolytic cell death, which together, can lead to antitumor immune responses (46, 47).

### Cell Lysis: Engineered

Other OVs have been engineered to induce cell death through mechanisms involving both virolysis and the introduction of suicide genes. Some adenoviruses have been modified to express HSV-1 TK. Once the virus targets the cancer cell using its specific natural or engineered tropism, it allows for a unique mechanism of cell killing when combined with thymidine analogs (e.g., acyclovir or ganciclovir). Unlike normal human TK, the HSV-1 TK can activate these thymidine analogs by converting them into monophosphates (48). These monophosphates are subsequently incorporated into the DNA of replicating cells which leads to chain termination and cell death. To further increase specificity, tumor-specific promoters are used to regulate the expression of HSV-1 TK. Clinical trials in which the osteocalcin promoter regulates HSV-1 TK expression have been used to target bone metastases. Similar to HSV-1 TK, another viral suicide gene, adenovirus death protein has been incorporated into OVs in preclinical studies to increase cell death (49). “Arming” OVs with suicide genes enhances their overall efficacy through increasing the ability of these viruses to directly kill cancer cells. Other OVs have been engineered to express pro-apoptotic molecules, such as TNF-related apoptosis-inducing ligand (TRAIL), which classically have been associated with apoptosis, but recently also shown to be involved in necroptosis (i.e., programmed necrosis) (50–53).

## Immune Activation

### Immune Responses: Natural

Beyond using the inherent lytic potential of OVs to provide direct killing of the cancer cell, viral infection of cancer cells elicits the standard antiviral immune response to clear the viral infection (Figure 3). In this process, it is proposed that antitumor responses can be revitalized or initiated by converting the environment from a suppressive into an inflamed tumor microenvironment. Briefly, viral infection results in the increase of pro-inflammatory cytokines, which recruit and activate both innate and adaptive immune cells. Further, viral infection results in the release of potent immune stimulators, toll-like receptor (TLR) ligands, which are critical for activating antigen-presenting cells (APCs), NK cells, and T cells. The combination of the release of cytokines and of TLR ligands as a result of infection of cancer cells with OVs is proposed to alleviate tumor-induced immune suppression. In addition, viral lysis leads to the release of intracellular tumor antigens that have not been presented and thus otherwise remain hidden to the immune system. Thus, oncolytic viral infection may release cancer antigens, including neoantigens (to which the immune system has not yet been tolerized) in the context of an inflammatory immune response, thus generating an effective antitumor response.

Mechanisms that some malignancies use to subvert the host immune system, in turn make them more susceptible to OVs. An example of this is interference with the type I IFN signaling pathway, which acts systemically to heighten antiviral and antitumor immune responses and locally to decrease cellular proliferation and increase p53, thereby activating the host apoptotic pathways (54). A number of cancers manipulate this pathway by decreasing type I IFN expression, decreasing receptor expression, or altering signaling downstream. While this leads to an optimal environment for cancer replication, it is also ideal for viral infection since in healthy tissues viruses are frequently cleared by the type I IFN responses. This, therefore, allows OVs, including vesicular stomatitis virus (VSV), vaccinia, Newcastle disease virus, mumps virus, alphaviruses, rabbit myxoma virus, and others to have specificity for these cells and the resulting microenvironment.

Additional factors have to be considered for oncolytic viral treatments. These include understanding the ability of a virus to hide from the immune system as well as possible antibodies generated against a virus since immunity may already exist to certain viruses. Some OVs are ones that humans are commonly exposed to, including adenovirus and poxviruses. Therefore, neutralizing antibodies may be present in the serum prior to treatment and further antibodies produced more rapidly based on immunological memory (55). However, exposure to and neutralizing antibodies against some OVs are less common (e.g., Seneca Valley virus). Such OVs provide an advantageous therapeutic window for treatment prior to immune neutralization of the virus (32).

### Immune Responses: Engineered

In addition to the immune-mediated mechanism of action for OVs that is a result of standard antiviral responses, OVs have been specifically engineered to further potentiate the immune response (Figure 3). To improve immune-mediated tumor destruction, OVs induce cancer cells to express pro-inflammatory cytokines, increase antigen presentation by cancer cells, and promote a more immunogenic form of cancer cell death. Cancer cells prevent immune destruction through altering the tumor microenvironment by recruiting immune suppressive cells and producing cytokines that limit antitumor responses. OVs specifically target cancer cells and are engineered to modify the suppressive tumor microenvironment. T-VEC was engineered with two copies of the human GM-CSF gene based on this idea. This immune stimulatory molecule can recruit professional APCs including dendritic cells, promote presentation of cancer antigens, lead to an influx and maturation of immune cells, and activate NK cells and tumor antigen-specific T cells (20, 56–58). Similar modifications have been made in adenoviruses and vaccinia virus (59, 60). In addition, adenoviruses have been engineered to enhance intrinsic antigen presentation by the cancer cell. Specifically, they have been modified to overexpress heat shock protein 70 (HSP70) in their target cells. This leads to increased intracellular protein trafficking to proteasomes, which directly leads to the increased availability of protein fragments for antigen presentation. HSP70 also has the unique characteristics of being directly associated with antigen presentation and allows for more peptides to be seen by APCs because these cells have an affinity for HSP70-linked peptides (61).

Natural serotype switching provides an advantageous method of treatment for viruses that possess multiple serotypes such as VSV and adenoviruses. However, serotype switching can also be mimicked through engineering of a virus to aid in immune system evasion and has been successfully done in measles virus (62). Other methods developed to avoid antibody neutralization include the encapsulation of OVs in polymer coatings to ensure viral replication and circulation (63, 64).

## Conclusion

Despite the immense progress evident in the development of OVs, it is important to note that substantial work remains in regards to understanding the mechanics and the ultimate potential of OVs. Genetic engineering to augment therapeutic efficacy still needs to be studied. Further, the proper administration and doses of different viruses should be investigated. As more information arises describing the use of new OVs, increases in biosafety procedures and protocols will also be a factor to consider. Clinical implications regarding the correct cancers to target and appropriate patients for whom to use oncolytic therapies will also be important to understand.

Oncolytic viruses used in therapy have had modest clinical success as stand-alone treatments for cancer. While efforts are being made to improve the overall efficacy of these viruses as individual therapies (including by engineering better OVs), combining OVs with currently approved cancer treatments may drastically improve therapy. Because of their unique mechanism of action, specific ability to target cancer cells, and good safety profile, OVs have been combined with many standard cancer therapies including surgery (NCT02714374), chemotherapy (NCT02779855), radiation therapy (NCT02453191), hormone modulators (NCT01867333), targeted therapies (NCT03088176), and even other immunotherapies (NCT02978625) (10, 65–71).

In addition, OVs have been combined with other OVs to enhance cancer cell lysis, improve targeting, and overcome immunity developed against multiple administrations of the same virus. Herpes virus combined with adenovirus in a pancreatic tumor model has been shown to improve the lytic release of adenovirus (72). Echoviral infection, which results in the upregulation of ICAM-1 (the cell entry receptor for Coxsackievirus) has been shown to improve efficacy of coxsackievirus (26). Further, the combination of vaccinia expressing the tumor antigens CEA and Muc1 (Panvac-v) and fowlpox virus expressing the same tumor antigens (Panvac-f) has been shown to improve direct lysis and immune-mediated tumor destruction (73–75).

Further, OVs have been combined with recently FDA-approved immunotherapies to enhance the immune-mediated mechanisms associated with tumor clearance. Specifically, talimogene laherparepvec (T-VEC), a herpes virus encoding GM-CSF, is being assessed for its ability to be combined with the immune checkpoint blockers ipilimumab (an antibody against CTLA-4) (NCT01740297) and pembrolizumab (an antibody against PD-1) (NCT02263508). Similarly, adenoviruses have been combined with checkpoint inhibitors in several ongoing studies in various malignancies including melanoma (NCT03003676), lung cancer (NCT02879760), and breast cancer (NCT03004183). The potential synergy from initiating immune responses with OVs and then blocking tumor immune suppression with immune checkpoint blockade represents an attractive therapy to generate effective antitumor responses. Such responses may be achieved in greater proportions of patients than either therapy has delivered alone. The emergence and imminent regulatory approval of chimeric antigen receptor (CAR)-modified T cells will lead to yet another potential combination therapy. CAR-T cells have been limited in their ability to treat solid tumors, likely secondary to poor trafficking to the immunosuppressive microenvironment (76). Early preclinical work, however, has established the potential benefits of this combination in solid tumors through viral modifications allowing for better tumor infiltration by and survival of CAR-T cells (77). Given the near limitless viral modifications that can be made to locally deliver specific genes and gene products to the tumor microenvironment, OVs have the potential to have an ever expanding role as an adjuvant to current and upcoming systemic immune and non-immune-mediated therapies.

Clinical trials have demonstrated that OVs are tolerable and their common side effects include those present in natural viral infections (e.g., fever, fatigue, and other flu-like symptoms) (78, 79). Improvement of OV specificity for cancer cells can be utilized to achieve the highest possible therapeutic efficacy while limiting side effects. Specifically, the antiviral immune response can at the same time limit the bioavailability and produce dose-limiting side effects, while both the antitumor and antiviral immune response can mediate therapeutic antitumor responses. Another safety consideration of using live virus for treatment is the possibility of transmission to others. However, transmissibility to others is limited because the majority of OVs are attenuated through extensive passaging, neutralized by healthy humans with preexisting antibodies and have reduced ability to spread through saliva and urine. However, precautions should always be taken, as with any live virus use (similar to those currently in place for live virus vaccinations).

Overall, OVs have shown the potential to be a very effective method of immunotherapy that efficiently and preferentially kills cancer cells. OVs have shown great successes as a treatment modality that both directly lyses cancer cells and elicits strong antitumor immune responses. With T-VEC producing significant results in terms of tumor regression in the United States, and with H101 approval in China, the use of OVs as an immunotherapeutic strategy has the potential to change the way we treat our patients. Numerous viruses exist as future therapeutic candidates allowing various cancers to be targeted for treatment. OVs have thus emerged as a fascinating approach to combat cancer and will only continue to improve with increased funding, preclinical studies, and clinical trials.

## Author Contributions

All authors contributed to writing and/or providing feedback on the manuscript.

## Conflict of Interest Statement

The authors declare that the research was conducted in the absence of any commercial or financial relationships that could be construed as a potential conflict of interest.
